# Methyltransferase Setdb1 Promotes Osteoblast Proliferation by Epigenetically Silencing Macrod2 with the Assistance of Atf7ip

**DOI:** 10.3390/cells11162580

**Published:** 2022-08-19

**Authors:** Lijun Zhang, Liqun Xu, Xiaoyan Zhang, Ke Wang, Yingjun Tan, Gaozhi Li, Yixuan Wang, Tong Xue, Quan Sun, Xinsheng Cao, Ge Zhang, Zebing Hu, Shu Zhang, Fei Shi

**Affiliations:** 1 The Key Laboratory of Aerospace Medicine, Ministry of Education, Air Force Medical University, Xi’an 710032, China; 2 State Key Laboratory of Space Medicine Fundamentals and Application, China Astronaut Research and Training Center, Beijing 100094, China; 3 Institute for Advancing Translational Medicine in Bone & Joint Diseases, School of Chinese Medicine, Hong Kong Baptist University, Hong Kong 999077, China

**Keywords:** Setdb1, cell proliferation, mechanical unloading, bone loss

## Abstract

Bone loss caused by mechanical unloading is a threat to prolonged space flight and human health. Epigenetic modifications play a crucial role in varied biological processes, but the mechanism of histone modification on unloading-induced bone loss has rarely been studied. Here, we discovered for the first time that the methyltransferase Setdb1 was downregulated under the mechanical unloading both in vitro and in vivo so as to attenuate osteoblast proliferation. Furthermore, we found these interesting processes depended on the repression of Macrod2 expression triggered by Setdb1 catalyzing the formation of H3K9me3 in the promoter region. Mechanically, we revealed that Macrod2 was upregulated under mechanical unloading and suppressed osteoblast proliferation through the GSK-3β/β-catenin signaling pathway. Moreover, Atf7ip cooperatively contributed to osteoblast proliferation by changing the localization of Setdb1 under mechanical loading. In summary, this research elucidated the role of the Atf7ip/Setdb1/Macrod2 axis in osteoblast proliferation under mechanical unloading for the first time, which can be a potential protective strategy against unloading-induced bone loss.

## 1. Introduction

Bone is a dynamic organ that continually undergoes bone remodeling. Bone remodeling occurs through a balance between osteoclast-mediated bone resorption and osteoblast-mediated bone formation. It can be regulated by many factors, such as hormones, cytokines, and mechanical stimulation [1]. Mechanical unloading, long space flights, and bed rest lead to disuse osteoporosis and increase the risk of fracture [2,3]. It is urgent to explore the potential molecular mechanism and provide a scientific basis for formulating more effective measures. Previous studies have reported that a reduced proliferation of osteoblasts played a key role in unloading-induced bone loss [4]. However, the mechanisms affecting unloading-induced bone loss have not been clearly elucidated.

Epigenetics is a common mechanism by which organisms respond to external and internal environmental cues through alterations in gene expression [5]. Histone methylation, as an epigenetic modification, occurs mainly on lysine (K) or arginine (R) residues of histone H3 and histone H4, which confers an active or repressive state of genes [6]. A series of histone methyltransferases and demethylases are characterized to regulate osteogenic activity and bone formation. The methyltransferase G9 promotes the proliferation and differentiation of calvarial osteoblasts, and Sox9-Cre/G9a ^flox/flox^ mice show a severe hypomineralization of the cranial fornix [7]. Furthermore, KDM4B recruits the CCAR1-Mediator complex by binding in the JmjC domain and induces chromatin structure change near osteoclast-related gene promoter by H3K9 demethylation [8]. Although there have been some advances in the changes of histone modification during bone formation, the relationship between histone modifying enzymes and osteoblast proliferation under mechanical unloading is not completely clear.

Setdb1, also termed ESET, is a histone methyltransferase and it participates in silencing genes through H3K9 trimethylation in the promoter region [9]. Setdb1 is involved in many pathological and physiological processes, such as carcinogenesis [10,11], cell lineage differentiation during embryogenesis [12], X chromosome inactivation [13], and transcriptional regulation [14,15]. Importantly, several studies have reported that Setdb1 plays a crucial role in bone formation. PPARγ is a key factor determining the fate of mesenchymal stem cells (MSCs) and promotes the differentiation of MSCs into adipocytes [16]. Setdb1 inhibits the transcriptional activation of PPARγ by regulating H3K9 methylation, thus inhibiting the differentiation of MSCs into adipocytes and promoting their differentiation into osteoblasts [17]. However, the role of Setdb1 in osteoblasts under mechanical unloading is still unclear. Our study focused on the effects of Setdb1 on osteoblasts, especially osteoblast proliferation.

In this study, we found that the methyltransferase Setdb1 was decreased in the tibial trabecular area of hindlimb unloading (HLU) mice, and bone-targeted si-Setdb1 attenuated bone formation. Consistent with this in vivo change, Setdb1 in MC3T3-E1 cells was downregulated in vitro under mechanical unloading and promoted osteoblast proliferation under both normal and unloading conditions. Furthermore, Setdb1 repressed the expression of Macrod2 by catalyzing the formation of H3K9me3 in the promoter region. Macrod2 was upregulated under mechanical unloading and suppressed osteoblast proliferation through GSK-3β/β-catenin signaling. In addition, Setdb1 was transferred from the nucleus to the cytoplasm under mechanical unloading, which was caused by a decrease in Atf7ip. In summary, our study reports for the first time that Setdb1 promotes osteoblast proliferation by regulating Macrod2 expression with the assistance of Atf7ip under mechanical unloading, which may aid in establishing a potential therapeutic strategy against osteoporosis.

## 2. Materials and Methods

### 2.1. Cell Culture

The preosteoblastic MC3T3-E1 cell line was purchased from the Cell Bank of the Chinese Academy of Sciences (Shanghai, China). MC3T3-E1 cells were cultured in an α-MEM medium containing 10% fetal bovine serum (Gibco, Waltham, MA, USA) and 1% penicillin/streptomycin (HyClone, Logan, UT, USA) in a cell incubator at 37 °C with 5% CO_2_. When cells were 90% confluent, three to six generations of cells in the logarithmic growth phase were selected for experiments. To study the localization of Setdb1, the proteasome inhibitor Bortezomib (20 nM for 40 h) (Topscience, Shanghai, China) was used to culture the cells.

### 2.2. Cell Transfection

A Lipofectamine 2000 kit (Invitrogen, Waltham, MA, USA) was used for cell transfection. siRNA targeting Setdb1, siRNA-Macrod2, or siRNA-Atf7ip (80 nM) and their negative controls were purchased from GenePharma (Shanghai, China) and used to transfect cells at 30–50% confluence according to the manufacturer’s instructions. pcDNA3.1-Setdb1(200 ng/μL) (Genechem, Shanghai, China), pcDNA3.1-Macrod2 (200 ng/μL) (Genepharma, Shanghai, China) and pcDNA3.1-Atf7ip (200 ng/μL) (GeneCreate, Wuhan, China) and their negative controls were used to transfect osteoblasts to induce overexpression. Supplementary Table S1 shows the sequences of the siRNAs and negative controls.

### 2.3. HLU Model

Male C57BL/6J mice for the HLU model were purchased from the Animal Center of Air Force Medical University (Xi’an, China). The male C57BL/6J mice at 6 months of age were maintained under standard conditions (22 °C, 12 h light/12 h dark cycle). The mice were hoisted by their tails, with the axis of the body at 30° to the ground for 21 days, so that the hindlimbs were suspended. The forelimbs touched the floor, which allowed the mice to move freely and obtain food and water. After the mice were anesthetized and euthanized, bilateral femurs and tibias were removed. Microcomputed tomography (µCT) and immunohistochemistry (IHC) were used for further analyses. All protocols were approved by the Experimental Safety Committee and Animal Care Committee of Air Force Medical University.

### 2.4. Targeted Silencing of Setdb1 Model

Male C57BL/6J mice were purchased from the Animal Center of Air Force Medical University (Xi’an, China) and maintained under standard conditions (22 °C, 12 h light/12 h dark cycle). The (AspSerSer)_6_-liposome system involving dioleoyl trimethylammonium propane (DOTAP)-based cationic liposomes attached to six repetitive sequences of aspartate, serine, and serine ((AspSerSer)_6_) delivers siRNAs specifically to bone-formation surfaces. Additionally, the (AspSerSer)_6_-liposome delivery systems were described in detail previously [18]. Four-week-old male mice were given two pulsed injections of the (AspSerSer)_6_-liposome system with si-Setdb1 or si-NC (10 mg/kg) every 2 weeks in 4 weeks [19]. After the mice were euthanized, the bilateral femurs and tibias were taken for bone analysis. Specifically, the right femurs were used for the three-point bending test, while the left femurs were harvested for micro-CT analysis and histological analysis. We referred to (AspSerSer)_6_ as (DSS)_6_ for short in the following. All protocols were approved by the Experimental Safety Committee and Animal Care Committee of Air Force Medical University.

### 2.5. 2D Clinorotation

Two-dimensional clinorotation (developed by the China Astronaut Research and Training Center, Beijing, China) is widely accepted to simulate microgravity in vitro on the ground. Approximately 1 × 10^5^ MC3T3-E1 cells were placed on coverslips. After cell adherence, the coverslips were inserted into chambers full of culture medium at a distance of 12.5 mm from the rotation axis. Next, the lids of the chambers were tightened after the bubbles had been completely removed. Finally, the chambers were placed into 2D clinorotation and rotated about the axis at 24 rpm. The same chambers for the control group were placed in an incubator at 37 °C.

### 2.6. Micro-CT Analysis

The left femurs and tibias of each mouse were fixed in 4% paraformaldehyde for 24 h and scanned via micro-CT branded as Siemens (Munich, Germany) or PerkinElmer (Waltham, MA, USA). The samples were scanned over a 360° rotation in increments of 0.5°. The region of interest (ROI) represented the microstructure of the femurs and tibias, measured in a 2.5 × 2.5 × 3 mm^3^ cube approximately 1.5 mm from the proximal epiphyseal plate. The following structural parameters in the ROI were analyzed: BMD, Tb. Th, BV/TV, and Tb. N. These data were used for blind analysis.

### 2.7. Three-Point Bending Test

The biomechanical properties of the femurs were evaluated by a three-point bending test using an electromechanical material testing machine (Bose, Framingham, MA, USA). The left femurs of mice were wrapped with gauze soaked in normal saline and stored at −80 °C. The femur samples were placed on a bending fixture with a span of 8 mm, and a load was applied perpendicular to the central axis of the femur at a speed of 0.02 mm/s until the fracture. Then, the length and width of the fracture site and the thickness of the bone cortex were measured with a vernier caliper. The maximum load (N), stiffness (N/mm), and elastic modulus (Gpa) were calculated according to the load-deflection curve.

### 2.8. qRT–PCR

Total RNA was extracted from cells with RNAiso Plus (TaKaRa, Tokyo, Japan). A Prime Script™ RT Master Mix reagent kit (TaKaRa, Tokyo, Japan) was used to convert the mRNA into cDNA. The expression of target genes was measured using a CFX96 real-time PCR detection system (BIO-RAD, Hercules, CA, USA) with SYBR Premix Ex Taq TM II (TaKaRa, Tokyo, Japan), and GAPDH was used as the endogenous control. Supplementary Table S2 shows the primer sequences.

### 2.9. Western Blotting

Cells were lysed with M-PER Mammalian Protein Extraction Reagent (Thermo Scientific, Waltham, MA, USA) containing 10% protease inhibitor (Roche, Basel, Switzerland) and were then ultrasonicated to extract protein. The concentration of each protein sample was measured by a bicinchoninic acid (BCA) protein assay kit (Thermo Scientific, Waltham, MA, USA). Loading buffer was added to the protein samples. Equal amounts of protein samples were added to NuPAGE™ Bis-Tris Protein Gels (Invitrogen, USA) and separated for 2 h. Proteins were transferred to polyvinylidene difluoride membranes in an ice bath, and then the membranes were sealed in 5% skim milk for 4 h at room temperature. The membranes were incubated with primary antibodies overnight at 4 °C. Primary antibodies specific for the following targets were used: PCNA (1:1000, Cell Signaling Technology, Boston, MA, USA), Setdb1 (1:1000, Cell Signaling Technology, Boston, MA, USA), H3K9me3 (1:1000, Cell Signaling Technology, Boston, MA, USA), p-GSK-3β (1:1000, Cell Signaling Technology, Boston, MA, USA), GSK-3β (1:1000, Cell Signaling Technology, Boston, MA, USA), β-Catenin (1:1000, Cell Signaling Technology, Boston, MA, USA), Macrod2 (1:800, Biorbyt, Cambridge, UK), c-Myc (1:1000, Cell Signaling Technology, Boston, MA, USA), Cyclin D1 (1:1000, Cell Signaling Technology, Boston, MA, USA), Lamin B1 (1:50,000, Proteintech, Wuhan, China), and GAPDH (1:1000, Cell Signaling Technology, Boston, MA, USA). Then, membranes were incubated with peroxidase-conjugated secondary antibody (1:5000, ZSGB-BIO, Beijing, China) for 1 h and developed with Super Signal West substrate (Thermo Fisher Scientific, Waltham, MA, USA). Densitometric analyses of the bands were conducted using ImageJ software.

### 2.10. Chromatin Immunoprecipitation (ChIP) Assay

Preosteoblastic MC3T3-E1 cells were transfected with siRNA-Setdb1. A chromatin immunoprecipitation (ChIP) assay was performed using a Simple ChIP Plus Enzymatic Chromatin IP Kit (Cell Signaling Technology, Boston, MA, USA) according to the manufacturer’s instructions. The antibodies used for immunoprecipitation were as follows: anti-Setdb1 (Cell Signaling Technology, Boston, MA, USA) and anti-H3K9me3 (Cell Signaling Technology, Boston, MA, USA). Precipitated DNA was analyzed via qRT–PCR. The primer sequences used for PCR are listed in Supplementary Table S3.

### 2.11. Immunoprecipitation Assay

After the corresponding treatment, the MC3T3-E1 cells were washed with phosphate-buffered saline (PBS). Total proteins were extracted from cell lysates and incubated with IgG (1 μg, Santa Cruz, Dallas, TX, USA) or Anti-Atf7ip (2 μg, Santa Cruz, Dallas, TX, USA) overnight at 4 °C, with an appropriate amount of extracted protein as the input control. Protein A/G Plus-Agarose was added to form immune complexes and incubated for 2 h at 4 °C. After washing, the Protein A/G Plus-Agarose beads were boiled for 5 min and centrifuged to collect the supernatants for Western blotting.

### 2.12. Histological Analysis

Both bone and other organs were fixed in 4% paraformaldehyde. Tibias and femurs were decalcified in 10% EDTA for 3 weeks. The decalcified specimens were dehydrated in gradient ethanol and embedded in paraffin. After paraffin embedding, sections with a thickness of 5 µm were created along the long axis of the tibia and placed on slides. The sections were placed in an incubator at 37 °C to dry for 72 h. Bone sections were subjected to H&E staining and Goldner’s trichrome staining according to the manufacturer’s protocol. The paraffin sections of the heart, liver, lung, spleen, and kidney were stained with H&E according to the standard protocol. For immunohistochemistry, the sections were incubated overnight with a primary anti-Setdb1 antibody (1:1000, Cell Signaling Technology, Boston, MA, USA) at 4 °C. After incubation with an HRP (horseradish peroxidase)-bound secondary antibody (Beyotime, Shanghai, China), DAB staining and hematoxylin staining (Beyotime, Shanghai, China) were performed. The sections were observed with a light microscope (Nikon, Tokyo, Japan).

### 2.13. Immunofluorescence

Cells were seeded on slides in a six-well plate. Cells at 80% confluence were washed gently with PBS and fixed at room temperature with 4% polyformaldehyde (MISHU, Xi’an, China) for 15 min. Cells were immersed in 0.5% Triton X-100 prepared in PBS at room temperature for 15 min and were then blocked with normal goat serum in a wet box at room temperature for 30 min. Next, the cells were incubated successively with primary anti-Setdb1 antibody (1:800, Cell Signaling Technology, Boston, MA, USA) overnight at 4 °C and with FITC-conjugated secondary antibody (Proteintech, Wuhan, China) in a wet box in the dark at 37 °C for 1 h. Nuclei were stained with DAPI for 5 min, and slides were then stored at 4 °C in the dark. A confocal microscope (Carl Zeiss, Oberkochen, Germany) was utilized to observe cells.

### 2.14. CCK-8

Cell proliferation was measured using a Cell Counting Kit-8 (MISHU, Xi’an, China). The cells were plated in 96-well plates at a density of 2000 cells/well, and every well contained 100 µL of the medium. CCK-8 enhancement solution (10 µL/well) was added to the 96-well plate, and the cells were then cultured in a cell incubator at 37 °C with 5% CO_2_ for 1 h. Next, the absorbance of the reaction solution at 450 nm was determined using a microplate reader.

### 2.15. EdU Labeling

The cells were placed on cover slides and then subjected to simulated microgravity or cell transfection. A BeyoClockTM EdU Cell Proliferation Kit with Alexa Fluor 594 was used for EdU labeling assays. A fresh culture medium containing 20 μM EdU was used to replace half of the original medium. Following a 2 h incubation in the incubator, osteoblasts were fixed in 4% paraformaldehyde (MISHU, Xi’an, China) for 15 min. Next, the cells were immersed in 0.3% Triton X-100 (Sigma, St. Louis, MO, USA) in PBS at room temperature for 15 min and then incubated with Alexa 594 at room temperature for 30 min. Finally, the osteoblasts were incubated with Hoechst 33,342 in PBS for 10 min at room temperature. The stained cells were observed with a confocal microscope (Carl Zeiss, Oberkochen, Germany). Quantification was achieved by evaluating the proportion of EdU-positive cells.

### 2.16. Luciferase Assay

Approximately 4 × 10^4^ MC3T3-E1 cells were placed on a 96-well plate. Then, the cells were co-transfected with TOPFlash plasmid (Beyotime, Shanghai, China) or FOPFlash plasmid (Beyotime, Shanghai, China) and si-Setdb1, si-Maacrod2 or pcDNA3.1-Setdb1 using Lipofectamine 2000 (Invitrogen, Waltham, MA, USA). After transfection for 36 h, the luciferase reporter gene activities were detected by the dual-luciferase assay system (Beyotime, Shanghai, China), according to the manufacturer’s protocols.

### 2.17. Statistical Analysis

All data are expressed as the means ± SDs of at least three independent experiments and were analyzed using SPSS 22.0 software. Student’s two-sided *t*-test was used to compare two groups, and one-way ANOVA followed by LSD’s multiple comparisons test was used to compare multiple groups. Repeated measures ANOVA were used in the CCK-8 assay. *p* < 0.05 was considered significant.

## 3. Results

### 3.1. Setdb1 Is Downregulated In Vivo and In Vitro under Mechanical Unloading

**Figure 1:**
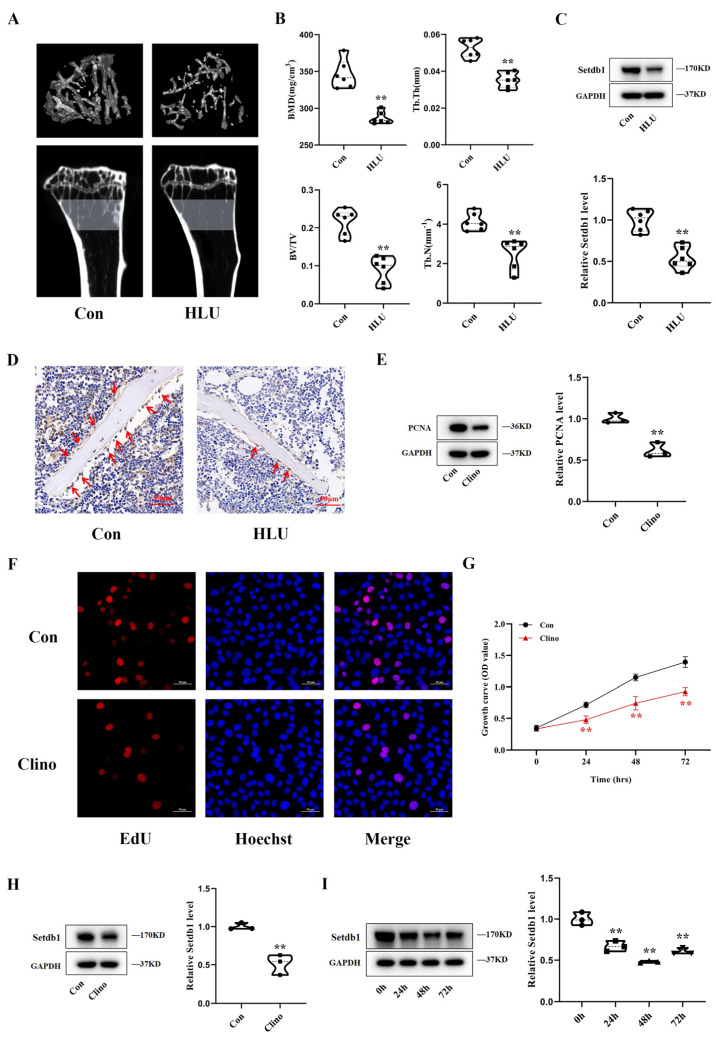
Setdb1 is downregulated in vivo and in vitro under mechanical unloading. (**A**) Representative images of micro-CT and three-dimension reconstruction for proximal tibias of mice in the Con and HLU group (*n* =  6). (**B**) The three-dimensional measurement of the bone mineral density (BMD), trabecular thickness (Tb. Th), trabecular number (Tb. N), and bone volume to total volume (BV/TV) in the ROI region of the proximal tibias of mice (*n* = 6). Student’s two-sided *t*-test. (**C**) The protein level of Setdb1 in bone tissues from each group (*n* = 6). Student’s two-sided *t*-test. (**D**) Representative images of Setdb1 immunohistochemical staining in the tibias of mice from each group (*n* = 6). Scale bar, 50 μm. (**E**) Western blotting analysis of PCNA protein levels in MC3T3-E1 cells (*n* = 3). Student’s two-sided *t*-test. (**F**) The EdU labeling assays of MC3T3-E1 cells in the Con and Clino group (*n* = 3). Scale bar, 50 μm. (**G**) Cell proliferation assessed by the CCK-8 assays from the Con and Clino group (*n* = 3). Repeated measures ANOVA. (**H**) Western blotting analysis of Setdb1 protein level in 48 h under clinorotation unloading (*n* = 3). Student’s two-sided *t*-test. (**I**) The changes in the Setdb1 protein level analyzed by Western blotting under clinorotation unloading for 24, 48, and 72 h (*n* = 3). One-way ANOVA was followed by LSD’s multiple comparisons test. ** *p* < 0.01 vs. the cells at 0 h.

To investigate the effect of mechanical unloading on osteoblasts, HLU mice and 2D clinorotation were used for in vivo and in vitro studies. HLU mice are a well-accepted model for simulating bone loss due to mechanical unloading. To construct the HLU model, we suspended the tails of C57BL/6J mice for 21 days. Micro-CT analysis demonstrated that the bone mineral density (BMD), the trabecular thickness (Tb. Th), the ratio of bone volume to total volume (BV/TV), and the trabecular number (Tb. N) decreased significantly in the HLU mice group (Figure 1A,B). This result was consistent with previous studies [20], which proved that the bone loss model was successful. Furthermore, the intraosseous amounts of Setdb1 protein were much lower in HLU mice than in Con mice (Figure 1C). Immunohistochemical staining clearly showed that the number of Setdb1-positive cells at bone-formation surfaces was lower in the tibias of HLU mice (Figure 1D and Figure S1A).

MC3T3-E1 cells were cultured in 2D clinorotation for 48 h to simulate mechanical unloading in vitro. The PCNA protein expression level remarkably decreased under mechanical unloading (Figure 1E). EdU labeling was used to assess the growth of the MC3T3-E1 cells. The growth of the MC3T3-E1 cells was significantly inhibited under mechanical unloading (Figure 1F and Figure S1B). In addition, the CCK-8 assay results were in good agreement with the EdU labeling assay (Figure 1G). Similarly, the expression of Setdb1 decreased under mechanical unloading in vitro (Figure 1H). Moreover, time-dependent experiments showed that Setdb1 expression was continuously downregulated under mechanical unloading and reached a low point at 48 h (Figure 1I). These results indicated that Setdb1 is downregulated in vivo and in vitro under mechanical unloading.

### 3.2. Setdb1 Regulates Osteoblast Proliferation and Bone Formation

**Figure 2:**
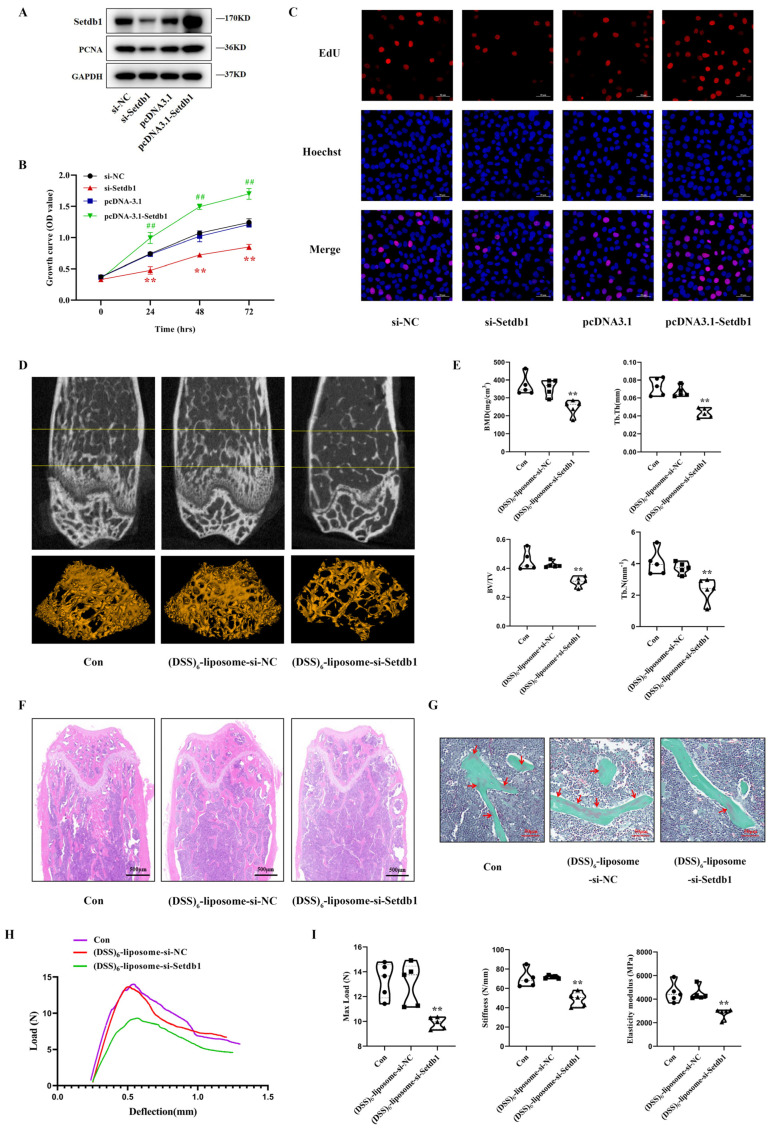
Setdb1 regulates osteoblast proliferation and bone formation. (**A**) The protein level of Setdb1 and PCNA in MC3T3-E1 cells by Western blotting after transfection of si-Setdb1, pcDNA3.1-Setdb1, or the corresponding controls (*n* = 3). (**B**) Cell proliferation evaluated by CCK-8 assays with the changes of Setdb1 (*n* = 3). Repeated measures ANOVA. ** *p* < 0.01 vs. the cells transfected with si-NC. ^##^ *p* < 0.01 vs. the cells transfected with pcDNA3.1. (**C**) The EdU labeling assays in MC3T3-E1 cells from each group (*n* = 3). Scale bar, 50 μm. (**D**) Representative images of micro-CT and three-dimension reconstruction for the distal femurs of mice in the indicated groups (*n* =  5). (**E**) The three-dimensional measurement of the bone mineral density (BMD), trabecular thickness (Tb. Th), the ratio of bone volume to total volume (BV/TV), and trabecular number (Tb. N) in the ROI region of the distal femurs of mice from each group (*n* =  5). One-way ANOVA followed by LSD’s multiple comparisons test. ** *p* < 0.01 vs. the mice injected with (DSS)_6_-liposome-si-NC. (**F**) H&E staining showing trabecular microarchitecture of the distal femurs from each group (*n* =  5). Scale bar, 500 μm. (**G**) Goldner’s trichrome staining showing newly formed bone in distal femurs of mice in the indicated groups (*n* =  5). Scale bar, 50 μm. (**H**) The load-deflection curves for the respective groups (*n* = 5). (**I**) The biomechanical parameters of femurs in the indicated groups (*n* =  5). One-way ANOVA followed by LSD’s multiple comparisons test. ** *p* < 0.01 vs. the mice injected with (DSS)_6_-liposome-si-NC.

To investigate the effect of Setdb1 on osteoblast proliferation, MC3T3-E1 cells were transfected with pcDNA3.1-Setdb1, si-Setdb1, or the corresponding controls. After transient transfection, the mRNA and protein expression of Setdb1 in MC3T3-E1 cells changed accordingly (Figure 2A and Figure S2A,B). Notably, the overexpression of Setdb1 in MC3T3-E1 cells significantly increased the PCNA protein level, while the PCNA expression decreased after the knockdown of Setdb1 (Figure 2A and Figure S2B). CCK-8 assays revealed that the knockdown of Setdb1 resulted in the significant inhibition of cell proliferation compared with that in the siRNA-NC group, whereas cell proliferation was promoted after the overexpression of Setdb1 (Figure 2B). Accordingly, an EdU labeling assay showed that Setdb1 knockdown retarded cell proliferation evidenced by an obvious decrease in EdU-positive cells, and the number of EdU-positive cells increased after the overexpression of Setdb1 (Figure 2C and Figure S2C). All these observations suggested that Setdb1 promotes osteoblast proliferation.

Given that disuse osteoporosis is a systemic bone disease, we further explored the effect of the systemically targeted silencing of Setdb1 in vivo on bone formation. To silence Setdb1 around the bone formation surface, si-Setdb1 or si-NC were encapsulated within the (DSS)_6_-liposome system approaching osteoblasts. Next, we evaluate the efficacy and safety of (DSS)_6_-liposome-si-Setdb1. The immunofluorescence staining showed co-staining of FAM-si-Setdb1 and Ocn-positive cells (osteoblasts) at bone-formation surfaces in (DSS)_6_–liposome–si-Setdb1-treated mice (Figure S2D). The qRT-PCR analysis revealed that bone-targeted si-Setdb1 effectively reduced the expression of Stedb1 in bone but has no significant effect on the other organs (Figure S2E). The hematoxylin and eosin (H&E) staining results showed no obvious tissue damage and pathological abnormalities in the (DSS)_6_-liposome-si-Setdb1 (Figure S2F). Furtherly, we further explored the effect of (DSS)_6_-liposome-si-Setdb1 on bone formation. The two-dimensional (2D) and three-dimensional (3D) image reconstruction of the distal femurs through micro-CT examination, showed that the trabecular structures in the (DSS)_6_-liposome-si-Setdb1 group were severely damaged, while these were more complete in the (DSS)_6_-liposome-si-NC group (Figure 2D). As shown by micro-CT analysis, we further found that the bone mineral density (BMD), trabecular thickness (Tb. Th), trabecular number (Tb. N), and the ratio of bone volume to total volume (BV/TV) in the (DSS)_6_-liposome-si-Setdb1 groups decreased significantly compared to the control group (Figure 2E). Consistent with the above results, the H&E staining of the distal femurs confirmed the low-bone-mass phenotype in the (DSS)_6_-liposome-si-Setdb1 group by quantifying the ratio of bone area to the total area (B.Ar/T.Ar) (Figure 2F and Figure S2G). Goldner’s trichrome staining showed that newly formed bone in the (DSS)_6_-liposome-si-Setdb1 group was less than that in the (DSS)_6_-liposome-si-NC group (Figure 2G). To further explore the biomechanical properties of the femurs, the three-point bending test on femurs was used to evaluate bone stiffness and strength. The load-deflection curves of each group were drawn according to the data (Figure 2H). The main structural parameters, maximum load, stiffness, and elastic modulus were significantly reduced in the (DSS)_6_-liposome-si-Setdb1 group compared to the control group (Figure 2I).

### 3.3. Setdb1 Silences Macrod2 Expression by Catalyzing the Formation of H3K9me3 in the Promoter Region

**Figure 3:**
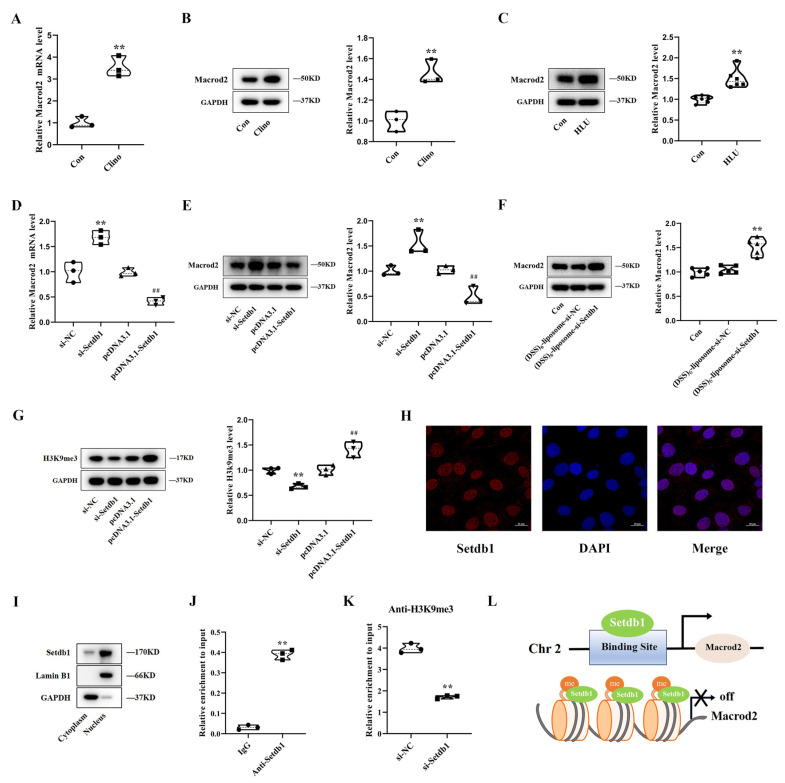
Setdb1 silences Macrod2 expression by catalyzing the formation of H3K9me3 in the promoter region. (**A**) The mRNA level of Macrod2 in MC3T3-E1 cells after exposure to clinorotation for 48 h by qRT-PCR (*n* = 3). Student’s two-sided *t*-test. (**B**) Western blotting analyses of Macrod2 protein level in MC3T3-E1 cells (*n* = 3). Student’s two-sided *t*-test. (**C**) Protein levels of Macrod2 in hindlimb bone tissues by Western blotting in the indicated groups (*n* = 6). Student’s two-sided *t*-test. (**D**) qRT-PCR analysis of Macrod2 mRNA levels in MC3T3-E1 cells transfected with si-Setdb1, pcDNA3.1-Setdb1, or the corresponding controls (*n* = 3). One-way ANOVA followed by LSD’s multiple comparisons test. ** *p* < 0.01 vs. the cells transfected with si-NC. ^##^ *p* < 0.01 vs. the cells transfected with pcDNA3.1. (**E**) Western blotting analyses of Macrod2 protein level in MC3T3-E1 cells after transfection of si-Setdb1, pcDNA3.1-Setdb1, or the corresponding controls. One-way ANOVA followed by LSD’s multiple comparisons test. ** *p* < 0.01 vs. the cells transfected with si-NC. ^##^ *p* < 0.01 vs. the cells transfected with pcDNA3.1. (**F**) Protein levels of Macrod2 in bone tissues by Western blotting in the indicated groups (*n* = 5). One-way ANOVA followed by LSD’s multiple comparisons test. ** *p* < 0.01 vs. the mice injected with (DSS)_6_-liposome-si-NC. (**G**) Protein levels of H3K9me3 by Western blotting from each group (*n* = 3). One-way ANOVA followed by LSD’s multiple comparisons test. ** *p* < 0.01 vs. the cells transfected with si-NC. ^##^ *p* < 0.01 vs. the cells transfected with pcDNA3.1. (**H**) The localization of Setdb1 was examined by immunofluorescence staining under normal conditions (*n* = 3). Scale bar, 20 μm. (**I**) The protein level of Setdb1 in cytoplasm and nucleus by Western blotting (*n* = 3). (**J**) ChIP-PCR assay illustrating Setdb1 association with the promoter of Macrod2 (*n* = 3). Student’s two-sided *t*-test. (**K**) ChIP-PCR assay indicating the enrichment of H3K9me3 by Setdb1 in Macrod2 promoter region (*n* = 3) Student’s 2-sided *t* test. (**L**) Schematic diagram of Setdb1 acting on macrod2 promoter region through formatting H3K9me3 (*n* = 3).

Setdb1 has been reported to contain a putative methyl-CpG binding domain that associated H3K9 methylation with DNA methylation [21,22], indicating that Setdb1 is involved in a variety of epigenetic modifications. The epigenetic mechanism of Setdb1 in osteoblast proliferation under mechanical unloading deserves further study. In our previous research, we found that many factors are sensitive to mechanical stimulation, among which Macrod2 contains CpG sites in the promoter region [23,24], which suggests that there would exist some Setdb1 binding sites in the promoter region of Macrod2. We further found that Setdb1 could bind to the DNA sequence of Macrod2 through Gene Transcription Regulation Database (GTRD). Western blotting assays revealed that Macrod2 was sensitive to mechanical stimulation and upregulated in clinorotation-unloading MC3T3-E1 cells (Figure 3A,B). Similarly, the intraosseous amounts of Macrod2 protein were much higher in HLU mice than in Con mice (Figure 3C). In addition, Macrod2 was reported to be negatively correlated with bone mineral density and cell proliferation [25,26]. Therefore, we considered Macrod2 an important candidate for Setdb1 target genes and hypothesized that Macrod2 could be regulated by Setdb1 through the formation of H3K9me3 in the promoter region, to affect osteoblasts proliferation under mechanical unloading.

To test this hypothesis, the qRT-PCR results showed that the mRNA level of Macrod2 increased markedly after Setdb1 knockdown and decreased after Setdb1 overexpression (Figure 3D). Similar results were observed at the protein level (Figure 3E). Macrod2 protein in bone tissues of the (DSS)_6_-liposome-si-Setdb1 mice was increased more than the negative control (Figure 3F). In addition, the expression level of H3K9me3 was also positively correlated with the expression of Setdb1 (Figure 3G). Through immunofluorescence and protein nucleocytoplasmic separation, we further confirmed that Setdb1 was mainly located in the nucleus in the normal condition (Figure 3H,I). The above results show that Setdb1, as a histone methyltransferase, affected the expression of H3K9me3 in the nucleus and the expression of Macrod2. To further determine the direct regulatory effect of Setdb1 on Macrod2, we conducted a ChIP assay. The ChIP-PCR assay revealed that Setdb1 bound directly to the promoter region of Macrod2 (Figure 3J), and after the knockdown of Setdb1, the occupancy of H3K9me3 in the promoter region of Macrod2 significantly decreased (Figure 3K). These results provided direct evidence that Setdb1 affects the expression of Macrod2 by maintaining the formation of H3K9me3 in its promoter region (Figure 3L).

### 3.4. Macrod2 Inhibits Osteoblast Proliferation in MC3T3-E1 Cells

**Figure 4:**
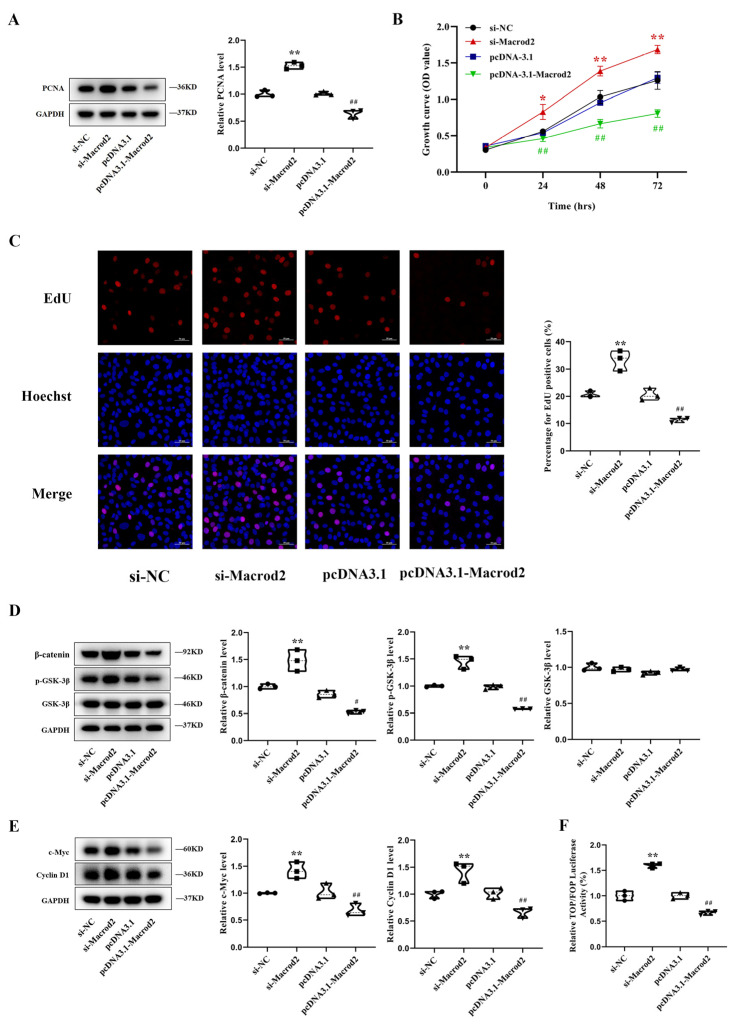
Macrod2 inhibits osteoblast proliferation in MC3T3-E1 cells. (**A**) Protein levels of PCNA in MC3T3-E1 cells by Western blotting in MC3T3-E1 cells transfected with si-Macrod2, pcDNA3.1-Macrod2, or the corresponding controls (*n* = 3). One-way ANOVA followed by LSD’s multiple comparisons test. (**B**) Cell proliferation as assessed by a CCK-8 assay in MC3T3-E1 cells in the indicated groups (*n* = 3). Repeated measures ANOVA. (**C**) The EdU positive cells showed by EdU labeling assays after treatment with si-Macrod2, pcDNA3.1-Macrod2, or the corresponding controls in MC3T3-E1 cells (*n* = 3). Scale bar, 50 μm. One-way ANOVA followed by LSD’s multiple comparisons test. (**D**) Western blotting analysis of β-catenin, p-GSK-3β, and GSK-3β in MC3T3-E1 cells (*n* = 3). One-way ANOVA followed by LSD’s multiple comparisons test. (**E**) Western blotting analysis for c-Myc and Cyclin D1 protein levels in MC3T3-E1 cells (*n* = 3). One-way ANOVA followed by LSD’s multiple comparisons test. (**F**) Dual luciferase assay showing the effect on TOP/FOP reporter activity in MC3T3-E1 cells transfected by si-Macrod2 or pcDNA3.1-Macrod2 (*n* = 3). One-way ANOVA followed by LSD’s multiple comparisons test. * *p* < 0.05 vs. the cells transfected with si-NC. ** *p* < 0.01 vs. the cells transfected with si-NC. ^#^ *p* < 0.05 vs. the cells transfected with pcDNA3.1. ^##^ *p* < 0.01 vs. the cells transfected with pcDNA3.1.

**Figure 5:**
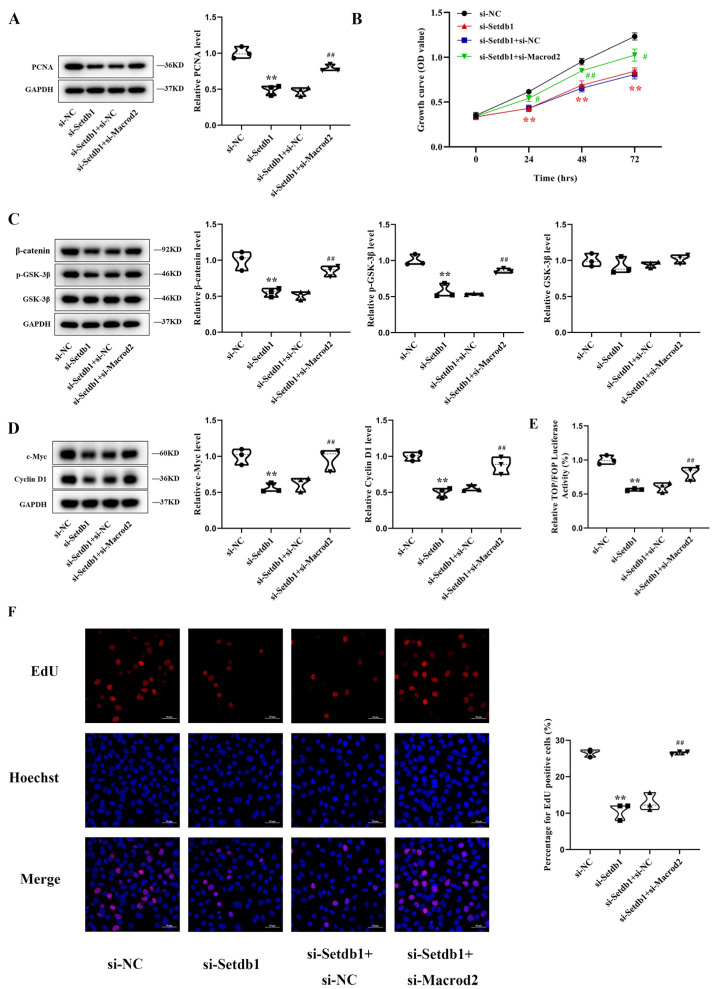
The knockdown of Macrod2 attenuates the inhibitory effect of Setdb1 knockdown on osteoblast proliferation in MC3T3-E1 cells. (**A**) Protein expression of PCNA in MC3T3-E1 cells by Western blotting after MC3T3-E1 cells cotransfected with si-Setdb1 alone or si-Setdb1 plus si-Macrod2 (*n* = 3). One-way ANOVA followed by LSD’s multiple comparisons test. (**B**) Cell proliferation as evaluated by a CCK-8 assay in MC3T3-E1 cells in the indicated groups (*n* = 3). Repeated measures ANOVA. (**C**) Western blotting analysis of β-catenin, p-GSK-3β, and GSK-3β in MC3T3-E1 cells (*n* = 3). One-way ANOVA followed by LSD’s multiple comparisons test. (**D**) Western blotting analysis for c-Myc and Cyclin D1 protein levels in MC3T3-E1 cells (*n* = 3). One-way ANOVA followed by LSD’s multiple comparisons test. (**E**) Measurement of TOP/FOP reporter activity in MC3T3-E1 cells (*n* = 3). One-way ANOVA followed by LSD’s multiple comparisons test. (**F**) The EdU labeling assays in MC3T3-E1 cells from each group (*n* = 3). Scale bar, 50 μm. One-way ANOVA followed by LSD’s multiple comparisons test. ** *p* < 0.01 vs. the cells transfected with si-NC. ^#^ *p* < 0.05 vs. the cells co-transfected with si-Setdb1 and si-NC. ^##^ *p* < 0.01 vs. the cells co-transfected with si-Setdb1 and si-NC.

To characterize the role of Macrod2 in osteoblast proliferation, we used siRNA and an overexpression vector to study the function of Macrod2. qRT–PCR and Western blotting analyses showed that Macrod2 was successfully knocked down or overexpressed after transient transfection (Figure S3A,B). Western blotting assays revealed that the knockdown of Macrod2 upregulated the PCNA protein level, whereas the overexpression of Macrod2 restrained this effect (Figure 4A). CCK-8 assays revealed that Macrod2 deficiency markedly promoted cell proliferation and that transfection with pcDNA3.1-Macrod2 inhibited cell proliferation compared with the control group (Figure 4B). Correspondingly, the EdU labeling assays showed that the knockdown of Macrod2 accelerated the growth of osteoblasts, whereas the overexpression of Macrod2 restrained this growth (Figure 4C). Macrod2 has also been reported to affect the activation of the GSK-3β/β-catenin signaling pathway by affecting GSK-3β [26,27]. Macrod2 deficiency inhibited the GSK-3β activity and increased β-catenin, which activates the GSK-3β/β-catenin signaling pathway. To examine the mechanism of Macrod2 function in MC3T3-E1 cells, proteins related to the GSK-3β/β-catenin signaling pathway were detected after alterations in Macrod2 expression. Western blotting assays showed that the knockdown of Macrod2 upregulated p-GSK-3β and β-catenin. Consistent with this, p-GSK-3β and β-catenin expression were decreased in Macrod2-overexpressing MC3T3-E1 cells. However, the total GSK level did not change regardless of the alterations in Macrod2 expression (Figure 4D). We also analyzed the expression of c-Myc and Cyclin D1, two target genes related to the cell proliferation of the GSK-3β/β-catenin signaling pathway. The knockdown of Macrod2 significantly increased the protein expression of c-Myc and Cyclin D1, whereas the overexpression of Macrod2 had the opposite effect (Figure 4E). To further determine the effect of Macrod2 on GSK-3β/β-catenin signaling activity, we tested the activity of the TOPFlash reporter in MC3T3-E1 cells. We found that knockdown of Macrod2 increased the luciferase activity, and the overexpression of Macrod2 decreased the activity (Figure 5F). Our results suggest that Macrod2 regulates osteoblast proliferation by inhibiting the activation of the GSK-3β/β-catenin signaling pathway in MC3T3-E1 cells.

### 3.5. Knockdown of Macrod2 Attenuates the Inhibitory Effect of Setdb1 Knockdown on Osteoblast Proliferation in MC3T3-E1 Cells

To confirm whether the regulatory effect of Setdb1 depends on Macrod2, MC3T3-E1 cells were cotransfected with si-Setdb1 and si-Macrod2 or its negative control. Western blotting analysis showed that after the cotransfection with siRNA-Setdb1 and si-Macrod2, the protein expression of PCNA was markedly increased, compared to that in the si-Setdb1 group (Figure 5A). The cotransfection with si-Setdb1 and si-Macrod2 partially blocked the si-Setdb1-induced reduction in cell proliferation capacity, based on CCK-8 assays (Figure 5B). Moreover, the reductions in the protein expression levels of p-GSK-3β and β-catenin in the si-Setdb1 group were partially reversed following the cotransfection with si-Macrod2. Consistent with the previous results, the total amount of GSK remained unchanged (Figure 5C). A similar trend was observed in the expression of c-Myc and Cyclin D1 (Figure 5D). In addition, si-Setdb1 reduced the activity of the GSK-3β/β-catenin signaling pathway, but the cotransfection of si-Macrod2 and si-Setdb1 partially blocked the reduction (Figure 5E). Similarly, si-Macrod2 significantly attenuated the decrease in EdU-positive cells induced by si-Setdb1 in MC3T3-E1 cells (Figure 5F). All these results suggest that Macrod2 is responsible for the regulatory effect of Setdb1 on osteoblast proliferation in MC3T3-E1 cells through the GSK-3β/β-catenin signaling pathway.

### 3.6. Setdb1 Blocks the Inhibition of Osteoblast Proliferation under Mechanical Unloading by Targeting Macrod2

**Figure 6:**
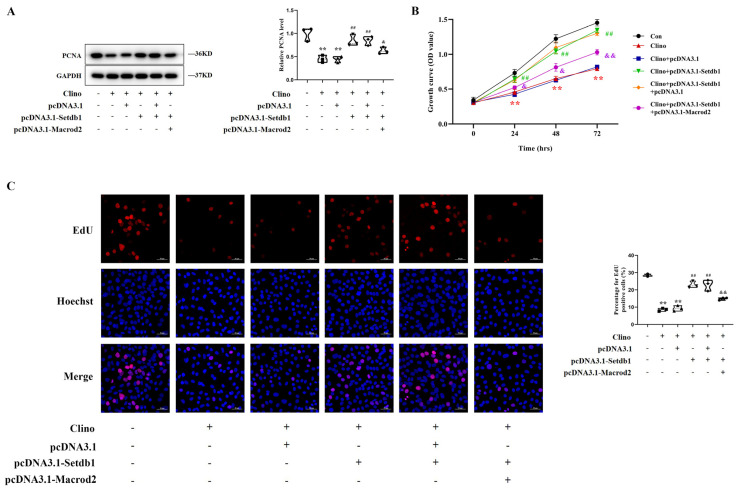
Setdb1 blocks the inhibition of osteoblast proliferation under mechanical unloading by targeting Macrod2. (**A**) PCNA protein expression measured by Western blotting after MC3T3-E1 cells were transfected with pcDNA3.1-Setdb1 alone or pcDNA3.1-Setdb1 plus pcDNA3.1-Macrod2 and subjected to clinorotation-unloading for 48 h (*n*  =  3). One-way ANOVA followed by LSD’s multiple comparisons test. (**B**) Cell proliferation assessed by a CCK-8 assay in MC3T3-E1 cells in the indicated groups (*n* = 3). Repeated measures ANOVA. (**C**) The EdU labeling assays by confocal microscope from each group (*n* = 3). Scale bar, 50 μm. One-way ANOVA followed by LSD’s multiple comparisons test. ** *p* < 0.01 vs. the control cells. ^##^ *p* < 0.01 vs. the cells transfected with pcDNA3.1 before unloading. ^&^ *p* < 0.05 vs. the cells co-transfected with pcDNA3.1 and pcDNA3.1-Setdb1 before unloading. ^&&^ *p* < 0.01 vs. the cells co-transfected with pcDNA3.1 and pcDNA3.1-Setdb1 before unloading.

We confirmed that Setdb1 was reduced in response to the mechanical unloading environment in MC3T3-E1 cells, and osteoblast proliferation was suppressed under mechanical unloading. Therefore, to study the role of Setdb1 in the inhibition of osteoblast proliferation under mechanical unloading, the MC3T3-E1 cells were transfected with pcDNA3.1-Setdb1 prior to culturing in 2D clinorotation for 48 h. Western blotting analysis showed that the overexpression of Setdb1 significantly attenuated the reduction in PCNA levels induced by mechanical unloading. Furthermore, the enhanced osteoblast proliferation induced by the overexpression of Setdb1 under mechanical unloading was reversed by Macrod2, evidenced by the level of PCNA protein expression (Figure 6A). Similarly, CCK-8 assays showed that Setdb1 partially rescued the inhibition of cell proliferation induced by mechanical unloading and that Macrod2 decreased this effect of Setdb1 (Figure 6B). In addition, the EdU labeling assay results were in good agreement with the CCK-8 assay results (Figure 6C). The experiment revealed that Setdb1 regulates osteoblast proliferation under mechanical unloading in a Macrod2-dependent manner.

### 3.7. Atf7ip Regulates the Nuclear Localization of Setdb1 and Exhibits a Similar Effect on Osteoblast Proliferation under Mechanical Unloading

**Figure 7:**
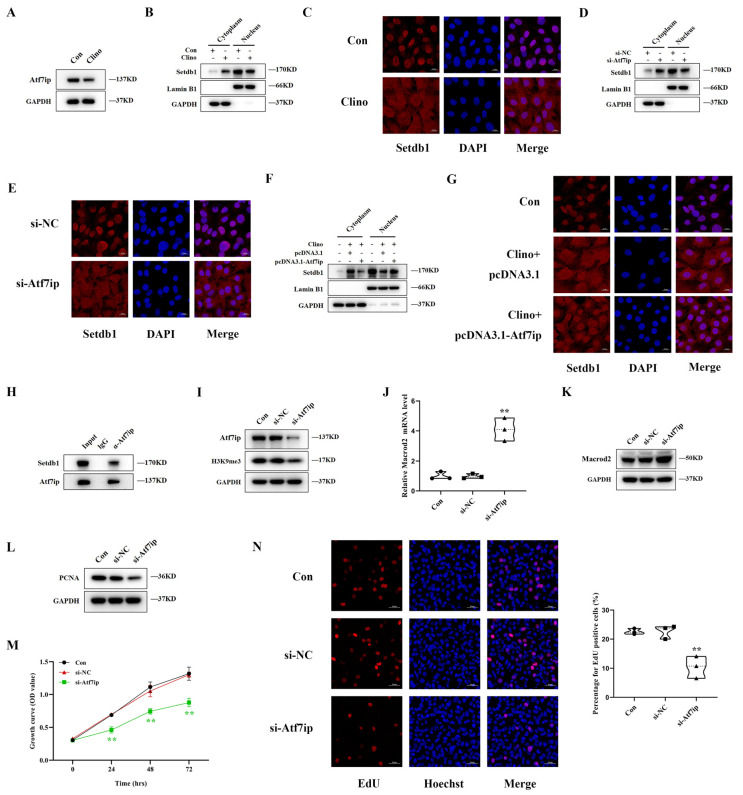
Atf7ip regulates the nuclear localization of Setdb1 and exhibits a similar effect on osteoblast proliferation under mechanical unloading. (**A**) Western blotting analysis of the Atf7ip protein level in 48 h under clinorotation unloading (*n* = 3). (**B**) Setdb1 expression in the nucleus and cytoplasm of MC3T3-E1 cells by Western blotting under clinorotation unloading for 48 h (*n* = 3). (**C**) The localization of Setdb1 examined by immunofluorescence staining (*n* = 3). Scale bar, 20 μm. (**D**) The protein level of Setdb1 in the nucleus and cytoplasm in MC3T3-E1 cells transfected by si-Atf7ip or its negative control by Western blotting (*n* = 3). (**E**) The localization of Setdb1 examined by immunofluorescence staining in MC3T3-E1 cells transfected by si-Atf7ip or its negative control (*n* = 3). Scale bar, 20 μm. (**F**)Western blotting analysis of Setdb1 in the nucleus and cytoplasm in MC3T3-E1 cells after treatment with pcDNA3.1-Atf7ip, or its negative control under clinorotation unloading for 48 h (*n* = 3). (**G**) Immunofluorescence staining showing the location of Setdb1 (*n* = 3). (**H**) The association between Atf7ip with Setdb1 was validated by Co-IP through anti-Atf7ip (*n* = 3). (**I**) The protein expression of Atf7ip and H3K9me3 in MC3T3-E1 cells transfected by si-Atf7ip and its negative control by Western blotting (*n* = 3). (**J**) qRT-PCR analysis of Macrod2 mRNA level in MC3T3-E1 cells transfected with si-Atf7ip, or the corresponding control (*n* = 3). One-way ANOVA followed by LSD’s multiple comparisons test. (**K**) Macrod2 protein expression by Western blotting (*n* = 3). (**L**) PCNA protein level by Western blotting in the indicated groups (*n* = 3). (**M**) Cell proliferation as evaluated by a CCK-8 assay in MC3T3-E1 cells in the indicated groups (*n* = 3). Repeated measures ANOVA. (**N**) The EdU labeling assays in MC3T3-E1 cells from each group (*n* = 3). Scale bar, 50 μm. One-way ANOVA followed by LSD’s multiple comparisons test. ** *p* < 0.01 vs. the cells transfected by si-NC.

Previous studies have reported that Atf7ip, as a binding partner of Setdb1, contributes to nuclear import and prevents the degradation of Setdb1 by proteases. We further explored whether Atf7ip regulates the localization of Setdb1 under mechanical unloading. Western blotting showed that the expression of Atf7ip decreased significantly under mechanical unloading (Figure 7A and Figure S4A). In the control group, Setdb1 was mainly located in the nucleus, but after mechanical unloading, Setdb1 was distributed in both the nucleus and cytoplasm by cell fractionation assays and immunofluorescence (Figure 7B,C). MC3T3-E1 cells were transfected with si-Atf7ip to knock down the expression of Atf7ip. qRT–PCR and Western blotting showed that the mRNA and protein levels of Atf7ip decreased significantly (Figure 7I and Figure S4B,F). After transfection with si-Atf7ip, the distribution of Setdb1 decreased in the nucleus and increased in the cytoplasm under a normal environment, similar to that under mechanical unloading (Figure 7D,E). In addition, we found that proteasome inhibitor bortezomib treatment increased the protein expression of Setdb1 in the nucleus (Figure S4C,D). To further confirm the role of Atf7ip in the localization of Setdb1 under unloading, we overexpressed Atf7ip under mechanical unloading. Our results showed that the distribution of Setdb1 in the nucleus increased (Figure 7F,G and Figure S4E). Moreover, through Co-IP experiments, we further confirmed that Atf7ip and Setdb1 formed a protein complex in MC3T3-E1 cells (Figure 7H). Therefore, we believe that the decrease in Setdb1 in the nucleus under mechanical unloading may be related to a decrease in Atf7ip.

In addition, the protein level of H3K9me3 decreased significantly after the Atf7ip knockdown (Figure 7I and Figure S4F). We also found that Atf7ip upregulated the mRNA and protein expression of Macrod2 (Figure 7J,K and Figure S4G). Furthermore, the knockdown of Atf7ip downregulated the PCNA protein level, evidenced by the Western blotting results (Figure 7L and Figure S4H). As shown by CCK-8 assays, the knockdown of Atf7ip significantly inhibited cell proliferation (Figure 7M). The EdU-positive cells decreased significantly in the si-Atf7ip group compared to the control group (Figure 7N). The knockdown of Atf7ip was shown to have a similar effect on H3K9me3 and osteoblast proliferation as the knockdown of Setdb1. This study indicated that Atf7ip regulates the nuclear localization of Setdb1 to regulate osteoblast proliferation under mechanical unloading.

## 4. Discussion

Bone loss caused by mechanical unloading has been a serious problem for prolonged space flights. Epigenetic modifications, including DNA methylation, histone modifications, and noncoding RNA, are involved in the regulation of multiple life processes. Our previous studies found that noncoding RNAs, such as miRNAs and lncRNAs [4,20,28,29,30,31], regulate the proliferation and differentiation of osteoblasts under mechanical unloading. However, the effect of histone modification on bone loss under mechanical unloading has rarely been reported. This study elucidates the role of the Atf7ip/Setdb1/Macrod2 signaling pathway in the process of osteoblast proliferation under mechanical unloading for the first time.

Setdb1 has been reported to be involved in bone remodeling and determines the fate of MSCs [16,17]. Setdb1(exons 15 and 16) ^Flox/Flox^ mice had fewer bone trabeculae, accompanied by a decrease in osteocalcin-positive cells and Alp-positive osteoblasts. The loss of Setdb1 suppressed the differentiation of MSCs into osteoblasts and the further mineralization of osteoblasts [32,33]. In addition, Setdb1 was found to regulate chondrocyte proliferation and cartilage mineralization during Meckel’s cartilage development [34]. However, the effect of Setdb1 on osteoblast proliferation under mechanical unloading has not yet been clearly elucidated, which was extremely essential for the occurrence of bone loss. In our study, we demonstrated that Setdb1 decreased in the tibial bone morphogenetic region of HLU mice and regulated the bone formation of mice. Consistent with this, clinorotation-unloading downregulated Setdb1 expression and inhibited osteoblast proliferation in vitro. Setdb1 promoted cell proliferation and blocked the degeneration proliferation caused by mechanical unloading.

Macrod2, an ADP-ribose glycohydrolase containing macro domains, plays an important regulatory role in various biological processes [35], such as tumorigenesis [36], embryonic development [37,38], metabolism balance [39,40] and various neurological diseases [41,42,43]. Interestingly, Pei YF [25] proposed that the Macrod2 gene is particularly important at Chromosome 20p12.1 and is a new candidate gene for bone regulation. They found that the bone mineral density in Macrod2 homozygous knockout mice was increased compared with the wild-type control, suggesting that Macrod2 may be involved in the bone regulatory pathway in an unknown way. In our study, we creatively proposed that Macrod2 is sensitive to mechanical stimulation in osteoblasts and is significantly upregulated in unloading osteoblasts and the tibial bone morphogenetic region of HLU mice. Previous studies have reported that the CpG site of Macrod2 is methylated, suggesting that it may be recognized by the methyl-CpG binding domain of Setdb1 [23,24]. Furthermore, Macrod2 was also reported to participate in the regulation of the Wnt signaling pathway, which reversed the inhibition of the GSK-3β mediated single ADP ribose. Similarly, a loss of Macrod2 inhibited GSK-3β activity and activated β-catenin signaling in hepatocellular carcinoma [26]. Here, we found that Macrod2 inhibited osteoblast proliferation by inhibiting the activation of the GSK3β/β-catenin signaling pathway, which partially contributed to the effect of Setdb1 on cell proliferation.

Furthermore, we explored the regulatory mechanism of Setdb1 under mechanical unloading. Atf7ip has been reported to coordinate DNA methylation and histone H3K9 methylation by interacting with Mbd1 and Setdb1 [13,44,45]. Atf7ip maintains Setdb1 stability and mediates the retention of Setdb1 in the nucleus, either by inhibiting its nuclear output or by promoting its nuclear input [46,47]. In our research, we revealed that Atf7ip was downregulated under mechanical unloading. Interestingly, the Setdb1 protein was widely distributed in the nucleus and cytoplasm under mechanical unloading, compared to that in the nucleus under normal conditions. After the knockdown of Atf7ip, the distribution of Setdb1 was also similar. It has been previously reported that a loss of Atf7ip leads to the repression of the Setdb1-regulated gene, accompanied by a reduction in H3K9me3, which is similar to the effects of Setdb1 inactivation [48,49]. Here, we revealed that the inhibition of osteoblast proliferation after the Atf7ip knockdown was similar to that after the Setdb1 knockdown. Furthermore, we discovered that the decrease in Atf7ip under mechanical unloading may lead to the transfer of Setdb1 to the cytoplasm. In contrast, previous studies have reported that a loss of Atf7ip did not affect the total protein level of Setdb1. However, our study showed that not only did the localization of Setdb1 change under mechanical unloading, but the total protein level of Setdb1 also decreased. We speculated that the decrease in Atf7ip may promote the degradation of Setdb1 by the proteasome. We further confirmed that treatment of the proteasome inhibitor Bortezomib attenuated the reduction in Setdb1 in the nucleus in the si-Atf7ip cells. However, the specific mechanism is still worth further study. In addition, our research focused on the effect of the Atf7ip/Setdb1 complex through histone methylation on cell proliferation. It is worthy of further study to explore the interaction between the Atf7ip/Setdb1 complex and Mbd1 through DNA methylation on bone formation.

## 5. Conclusions

In conclusion, this study uncovered an unloading-sensitive methyltransferase Setdb1, which is an important regulator of osteoblast proliferation. Setdb1, with the assistance of Atf7ip, regulated osteoblast proliferation, which depended on regulating Macrod2 expression by catalyzing H3K9me3 in the promoter region under mechanical unloading. Our research demonstrates that the functional promotion of Setdb1 effectively attenuates the restriction of mechanical unloading, suggesting that therapeutic approaches targeting Setdb1 may be helpful for enhancing bone formation and may be protective strategies against mechanical loading-induced bone loss.

## Data Availability

Not applicable.

## Supplementary Materials

The following supporting information can be downloaded at: https://www.mdpi.com/article/10.3390/cells11162580/s1. Figure S1: Statistical analysis of the number of Setdb1 positive osteoblasts per bone surface and percentage for EdU positive cells; Figure S2: Setdb1 regulates osteoblast proliferation and bone formation; Figure S3: the expression of Macrod2 in MC3T3-E1 cells by RT–qPCR and Western blotting; Figure S4: Atf7ip regulates the nuclear localization of Setdb1 and exhibits a similar effect on osteoblast proliferation under mechanical unloading. Table S1: RNA oligo sequences for transfection; Table S2: Primer sequences used for qRT–qPCR; Table S3: Primer sequences for ChIP-PCR assay.
