# Supplementary material for: Methyltransferase Setdb1 Promotes Osteoblast Proliferation by Epigenetically Silencing Macrod2 with the Assistance of Atf7ip
Source: Cells. 2022 Aug 19;11(16):2580. doi: 10.3390/cells11162580 (PMC9406310; doi:10.3390/cells11162580)
Supplement: Supplementary file 1 [file cells-11-02580-s001.zip › Supplementary Material.pdf]

Supplementary Materials for

Methyltransferase Setdb1 Promotes Osteoblast Proliferation by Epigenetically Silencing Macrod2 with the Assistance of Atf7ip

Lijun Zhang [1,†], Liqun Xu [1,†], Xiaoyan Zhang [1,†], Ke Wang [1], Yingjun Tan [2], Gaozhi Li [1], Yixuan Wang [1], Tong Xue [1], Quan Sun [1], Xinsheng Cao [1], Ge Zhang [3], Zebing Hu [1], Shu Zhang [1,*,‡], and Fei Shi [1,*,‡]

[1] The Key Laboratory of Aerospace Medicine, Ministry of Education, Air Force Medical University, Xi'an 710032, China
[2] State Key Laboratory of Space Medicine Fundamentals and Application, China Astronaut Research and Training Center, Beijing 100094, China
[3] Institute for Advancing Translational Medicine in Bone & Joint Diseases, School of Chinese Medicine, Hong Kong Baptist University, Hong Kong 999077, China
* Correspondence: shuzhang@fmmu.edu.cn (S.Z.); shifei719@fmmu.edu.cn (F.S.); Tel.: 86-29-8471-1231 (S.Z.)
† These authors contributed equally to this work.
‡ These authors contributed equally to this work.

Table S1 RNA oligo sequences for transfection

Name	Sequence (5' - 3')
**siRNA-Setdb1 sense**	CACUCAGUCAGAGCUUUAUTT
**siRNA-Setdb1 antisense**	AUAAAGCUCUGACUGAGUGTT
**siRNA-control sense**	UUCUCCGAACGUGUCACGUTT
**siRNA-control antisense**	ACGUGACACGUUCGGAGAATT
**siRNA-Macrod2 sense**	CCCUAAUGGUUGGAGAAAUTT
**siRNA-Macrod2 antisense**	AUUUCUCCAACCAUUAGGGTT
**siRNA-Atf7ip sense**	CCAUCAGGCGUGCCCACAATT
**siRNA-Atf7ip antisense**	UUGUGGGCACGCCUGUGGTT

Abbreviations: Setdb1, SET domain, bifurcated 1;

Table S2 Primer sequences used for RT-qPCR

Gene		Sequence (5' - 3')
**GAPDH**	F	TGTGTCCGTCGTGGATCTGA
	R	TTGCTGTTGAAGTCGCAGGAG
**Setdb1**	F	AGCTCCTGCCGAGACTTCATAGAG
	R	GCTGCCATCCACCTCTTCAACTC
**Macrod2**	F	CGGAGGAGGAGGTGTGGATGG
	R	CAGTCTCACAGCCGTTCAGGTTC
**Atf7ip**	F	GTGCGACAACTGCTGGAGTCC
	R	GTGGCGTGACTGGCTGAAGAC

Abbreviations: GAPDH, glyceraldehyde 3-phosphate dehydrogenase; Setdb1, SET domain, bifurcated 1; Macrod2, MACRO domain-containing protein 2; Atf7ip, activating transcription factor 7-interacting protein 1; RT-qPCR, reverse transcription quantitative real-time polymerase chain reaction.

Table S3 Primers for ChIP assay

Name		Sequence (5' - 3')
**The promoter region of Macrod2**	F	CAGAGCCTTTGCACTTTTCCCTC
	R	CTTTAAGCTGCAAGTTCCCCAGG

Abbreviations: ChIP: chromatin immunoprecipitation

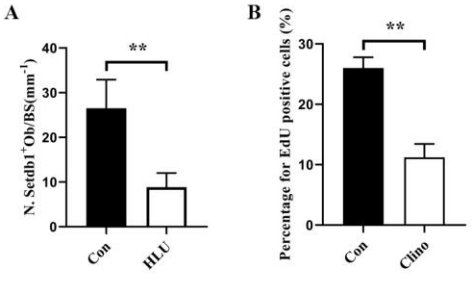

Figure S1 (A) Statistical analysis of the number of Setdb1 positive osteoblasts per bone surface in the femurs (n=6). Student’s 2-sided *t* test. **(B)** Statistical analysis of percentage for EdU positive cells (n=3). Student’s 2-sided *t* test. $**P < 0.01$ vs. control.

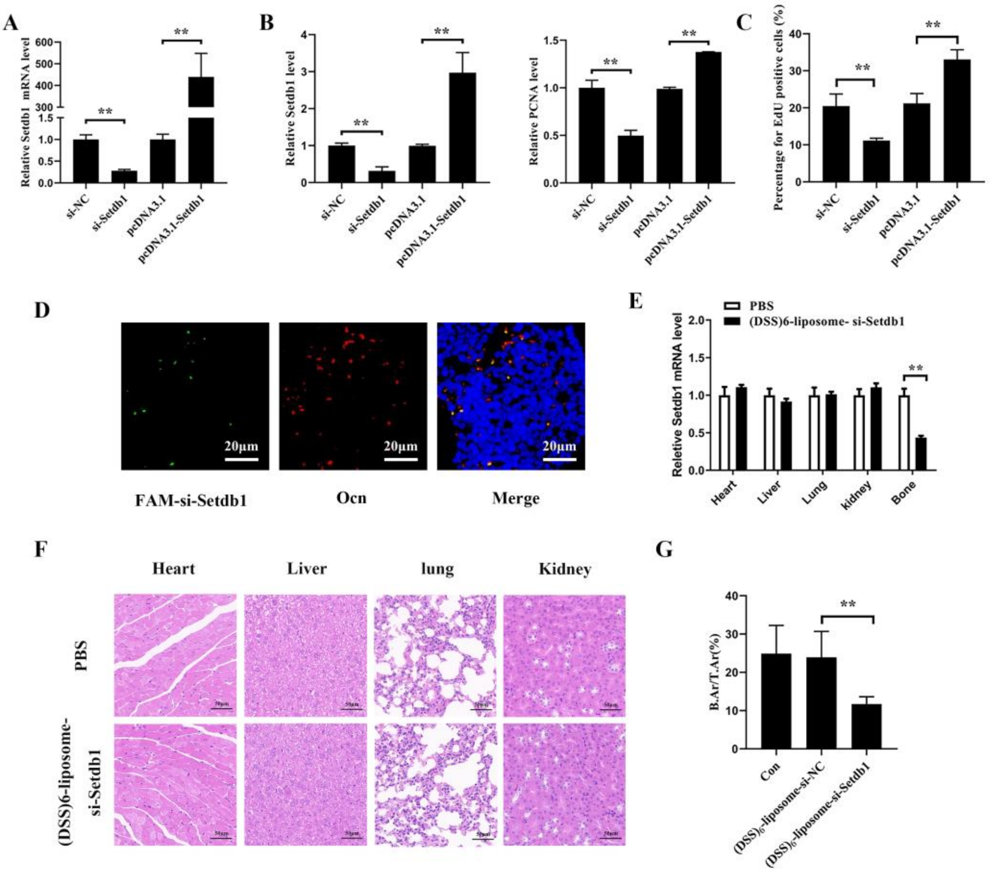

Figure S2 (A) The mRNA expression of Setdb1 in MC3T3-E1 cells transfected by si-Setdb1, pcDNA3.1-Setdb1 or the corresponding controls (n=3). One-way ANOVA followed by LSD's multiple comparisons test. **(B)** The protein expression of Setdb1 and PCNA in MC3T3-E1 cells transfected by si-Setdb1, pcDNA3.1-Setdb1 or the corresponding controls (n=3). One-way ANOVA followed by LSD's multiple comparisons test. **(C)** Statistical analysis of percentage for EdU positive cells (n=3). One-way ANOVA followed by LSD's multiple comparisons test. **(D)** Representative images of immunofluorescence staining in the femurs of mice. The si-Setdb1 was labeled with FAM (green, left column). Immunofluorescence staining of Ocn was performed to detect osteoblasts (red, middle column). Merged images with DAPI staining showed co-staining of si-Setdb1 and osteoblasts. Scale bar, 20μm. **(E)** The mRNA expression of Setdb1 in different tissues (heart, liver, lung, kidney and bone) three days after a single injection of (DSS)$_6$-liposome-si-Setdb1 or PBS (n=5). Student's 2-sided *t* test. **(F)** Representative images of H&E staining for heart, liver, lung and kidney were shown three days after a single injection of (DSS)$_6$-liposome-OGRU-si-Setdb1 or PBS (n=5). Scale bars, 50μm. **(G)** Quantitative analysis of the bone area/total area in the distal femurs of mice in the indicated groups (n=5). One-way ANOVA followed by LSD's multiple comparisons test. **$P < 0.01$ vs. control.

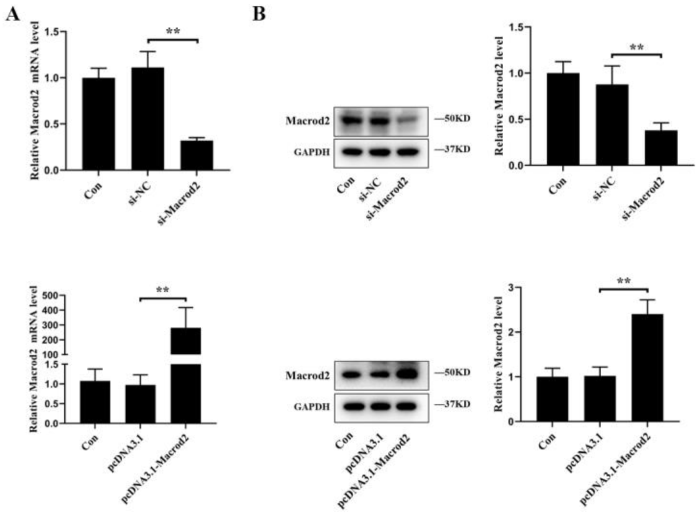

Figure S3 (A) qRT-PCR analysis of Macrod2 mRNA levels in MC3T3-E1 cells transfected with si-Macrod2, pcDNA3.1-Macrod2, or the corresponding negative control (n=3). **(B)** Western blotting analysis of Macrod2 protein levels in MC3T3-E1 cells transfected with si-Macrod2, pcDNA3.1-Macrod2 or its negative control by western blotting. (n=3). One-way ANOVA followed by LSD's multiple comparisons test. ***P* < 0.01 vs. control.

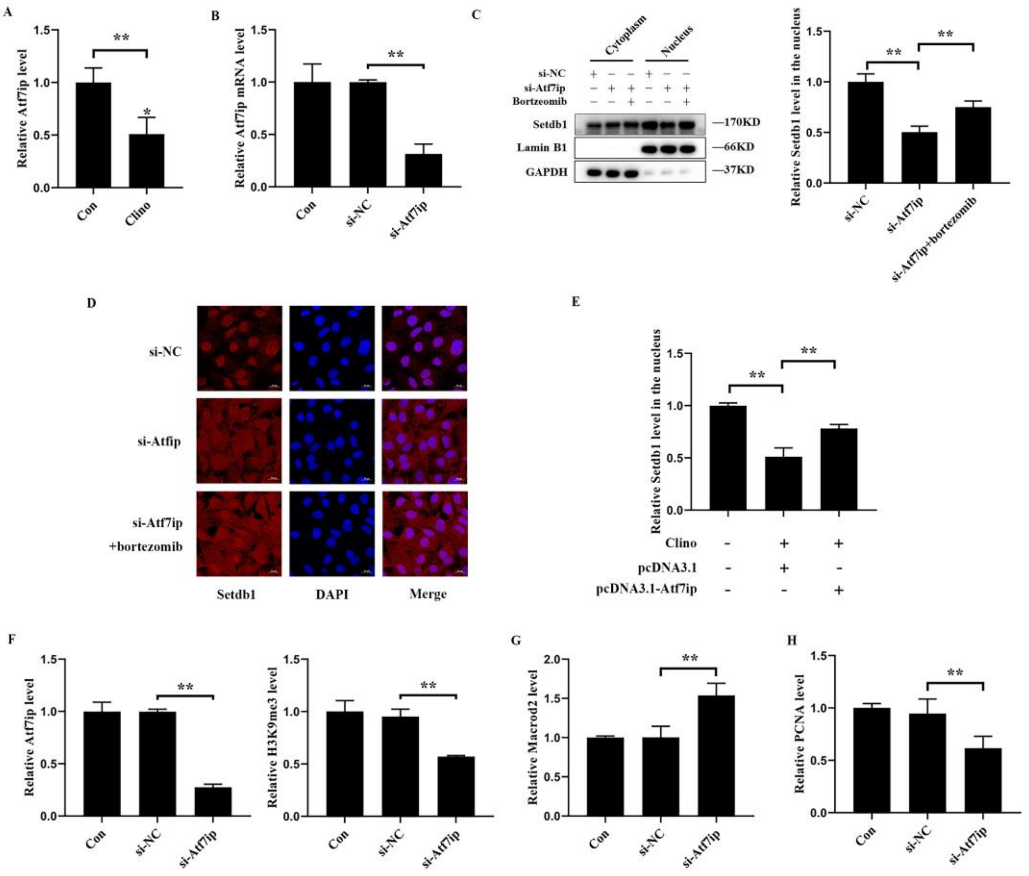

Figure S4 **(A)** Western blotting analysis of Atf7ip protein level in 48h under clinorotation unloading (n=3). Student's 2-sided *t* test. **(B)** The mRNA expression of Atf7ip in MC3T3-E1 cells transfected by si-Atf7ip or its negative control (n=3). One-way ANOVA followed by LSD's multiple comparisons test. **(C)** Western blotting analysis of Setdb1 in the nucleus of MC3T3-E1 cells after treatment with bortezomib (n=3). One-way ANOVA followed by LSD's multiple comparisons test. **(D)** The localization of Setdb1 examined by immunofluorescence staining under normal conditions (n=3). **(E)** Western blotting analysis of Setdb1 in the nucleus and cytoplasm in MC3T3-E1 cells after treatment with pcDNA3.1–Atf7ip, or its negative control under clinorotation unloading for 48h (n=3). One-way ANOVA followed by LSD's multiple comparisons test. **(F)** Western blotting analysis of Atf7ip and H3K9me3 protein levels in MC3T3-E1 cells transfected with si-Atf7ip, or its negative control (n=3). One-way ANOVA followed by LSD's multiple comparisons test. **(G)** The protein level of Macrod2 in MC3T3-E1 cells transfected by si-Atf7ip or its negative control by western blotting (n=3). One-way ANOVA followed by LSD's multiple comparisons test. **(H)** Western blotting analysis of PCNA protein levels in MC3T3-E1 cells transfected with si-Atf7ip, or its negative control (n=3). One-way ANOVA followed by LSD's multiple comparisons test. ***P* < 0.01 vs. Control
